# Advances in Imaging-Based Machine Learning and Therapeutic Technology in the Management of Retinal Diseases

**DOI:** 10.3390/medicina60111794

**Published:** 2024-11-01

**Authors:** Joshua Ong, Jay Chhablani

**Affiliations:** 1 Department of Ophthalmology and Visual Sciences, University of Michigan Kellogg Eye Center, Ann Arbor, MI 48105, USA; ongjo@med.umich.edu; 2 Department of Ophthalmology, University of Pittsburgh School of Medicine, Pittsburgh, PA 15213, USA

Retinal conditions like age-related macular degeneration (AMD), diabetic retinopathy, central serous chorioretinopathy (CSCR), and retinal vein occlusion can drastically affect a patient’s quality of life. However, advances in retinal technology are changing the landscape of how we diagnose, treat, and manage these diseases, opening doors to more personalized and effective care. This Special Issue in *Medicina* presents papers that cover the various technologies that are revolutionizing diagnosis, management, and treatment in retinal care.

Machine learning, a subset of artificial intelligence, has become a revolutionary technology in the realm of the retina. This was evidenced by IDx-DR becoming the first autonomous artificial intelligence system approved by the Food and Drug Administration (FDA), which was developed for the diagnosis of diabetic retinopathy [1]. In this Special Issue, Borrelli et al. discuss the advances in deep learning for AMD [2]. The authors describe the ability of deep learning to identify certain biomarkers that can help identify individuals at risk for transitioning to neovascular AMD. The paper discusses various techniques, such as hybrid modeling approaches that integrate radiomics, demographic data, and visual acuity with deep learning to predict exudation in patients with either early or intermediate age-related macular degeneration [2,3]. The authors also discuss their own deep learning work in AMD with optical coherence tomography (OCT) imaging by producing automated segmentation of intraretinal/subretinal fluid (IRF/SRF) and neovascular pigment epithelial defect (PED) [2,4]. As the field of retina has a large foundation in imaging, the integration of deep learning and ophthalmic imaging is an exciting prospect for future retinal care.

Deep learning-based care is a step toward providing highly personalized care for patients. The shift toward personalized medicine is an exciting development in all fields of medicine, including retina. A part of personalized medicine in ophthalmic health includes developing and investigating various treatment approaches, as well as understanding the interplay between overall systemic health and ocular health [5]. Singh et al. discuss exciting new developments in nanotherapeutics for AMD with a focus on pathophysiology and targeted processes for therapeutic development [6]. The authors discuss the role of various processes in AMD pathogenesis, including inflammation, oxidative stress, and bioactive lipids [6]. In addition to novel targets, the authors also explore the various delivery systems to help with the treatment of AMD. These techniques include employing nanocarriers to increase solubility and bioavailability for topical medications that may be helpful in providing different therapeutic options for patients [6]. In addition to topical therapeutics, this Special Issue also explores laser technology for retinal diseases. In their study, Ruggeri et al. investigate the changes in choroidal and choriocapillaris in eyes with CSCR following either subthreshold micropulse laser therapy or photodynamic therapy. The authors employ both OCT and OCT angiography (OCTA) in their analysis. While there were various differences between the two groups, the authors found that both therapies improve both functional and structural biomarkers in CSCR [7]. This research offers exciting insights into both treatment and choroidal biomarkers for CSCR progression and management.

Ultimately, the future of retina is promising and filled with various technological advances to improve care. This Special Issue explores these insights within this field to optimize care and quality of life for patients with retinal diseases. Future research in these areas will be critical to continue improving care and advancing technology for patients worldwide.
